# Multi-Omics Analysis Revealed Increased De Novo Synthesis of Serine and Lower Activity of the Methionine Cycle in Breast Cancer Cell Lines

**DOI:** 10.3390/molecules28114535

**Published:** 2023-06-03

**Authors:** Monika Pankevičiūtė-Bukauskienė, Valeryia Mikalayeva, Vaidotas Žvikas, V. Arvydas Skeberdis, Sergio Bordel

**Affiliations:** 1 Institute of Cardiology, Lithuanian University of Health Sciences, LT 50162 Kaunas, Lithuania; 2 Institute of Pharmaceutical Technologies, Lithuanian University of Health Sciences, LT 50162 Kaunas, Lithuania; 3 Institute of Sustainable Processes, University of Valladolid, Dr. Mergelina s/n, 47011 Valladolid, Spain

**Keywords:** breast cancer, genome-scale metabolic model, drug target

## Abstract

A pipeline for metabolomics, based on UPLC-ESI-MS, was tested on two malignant breast cancer cell lines of the sub-types ER(+), PR(+), and HER2(3+) (MCF-7 and BCC), and one non-malignant epithelial cancer cell line (MCF-10A). This allowed us to quantify 33 internal metabolites, 10 of which showed a concentration profile associated with malignancy. Whole-transcriptome RNA-seq was also carried out for the three mentioned cell lines. An integrated analysis of metabolomics and transcriptomics was carried out using a genome-scale metabolic model. Metabolomics revealed the depletion of several metabolites that have homocysteine as a precursor, which was consistent with the lower activity of the methionine cycle caused by lower expression of the AHCY gene in cancer cell lines. Increased intracellular serine pools in cancer cell lines appeared to result from the over-expression of PHGDH and PSPH, which are involved in intracellular serine biosynthesis. An increased concentration of pyroglutamic acid in malignant cells was linked to the overexpression of the gene CHAC1.

## 1. Introduction

The deregulation of metabolism has been known to be a characteristic of cancer cells since the work of Warburg, almost a century ago [1]. In their influential review, Hanahan and Weinberg [2] listed “deregulation of cellular energetics” among the emerging hallmarks of cancer. In a recent update by Hanahan [3], this hallmark was renamed “deregulation of cellular metabolism”. This change of name illustrates the fact that the metabolic changes that characterize cancer cells go well beyond the Warburg effect [4,5,6,7].

Metabolism is a complex system that emerges from the interplay of metabolic enzymes and the small molecules (metabolites) transformed by these enzymes. Metabolism can be studied from different angles, depending on which experimental techniques are used. Proteomics and transcriptomics enable the measurement of changes in enzyme concentrations (directly, using proteomics, or indirectly, using transcriptomics), fluxomics enables the measurement of reaction rates (for example, using ^13^C-labeled substrates) and metabolomics enables measurement of the concentrations of intracellular metabolites. The relationships between these three types of variables (enzyme levels, metabolic fluxes and metabolite concentrations) can be represented using so called genome-scale metabolic models (GSMMs) [8]. These models have been used to identify putative drug targets specific to different cancer cell lines [9,10], and, in combination with gene expression data, have revealed higher activity of the enzymes involved in the degradation of branched-chain amino acids, which was confirmed at the protein level for two cancer cell lines: MCF-7 and BCC [11].

However, to date, it is not possible to establish a direct relationship between enzyme levels, metabolic fluxes and metabolite concentrations. It has been shown that most of the changes in the expression levels of metabolic genes (those coding metabolic enzymes) do not result in concomitant changes in metabolic fluxes [12], and the transcriptional changes in just a few enzymes are enough to explain the rearrangement of metabolic fluxes within the cell [13,14]. A theoretical explanation for the mentioned observations was also proposed [15]. As perturbations in metabolic fluxes cannot be fully inferred from observed changes in enzyme concentrations, direct measurements of metabolic fluxes are necessary in order to understand the metabolism of cancer cells. In previous articles, we have focused on studying the metabolism of breast cancer cell lines (MCF-7 and BCC) from the point of view of metabolic fluxes, using ^13^C-labeled substrates and mass spectroscopy. These studies revealed a key role of lipid metabolism in fighting oxidative stress [16], and the fact that leucine degradation is at the source of a large fraction of the mevalonate that fuels cholesterol synthesis in malignant breast cancer cells [17].

There is also no direct relationship between metabolic fluxes and internal metabolite concentrations. Indeed, the methods used to infer metabolic flux distributions in steady state [18] do not require the quantification of metabolite concentrations, but just their mass spectra (reflecting their ^13^C labeling pattern). The same metabolic flux distribution (characterized by the steady state of each internal metabolite) could be achieved at different steady state concentrations of internal metabolites.

Internal concentrations of metabolites could have a key role in cancer progression by interacting with signaling pathways, and could also be used as informative biomarkers to assess malignancy [19]. The first oncometabolite discovered was 2-hydroxyglutarate [20], a rare metabolite found in high concentrations in gliomas. Since 2010, metabolomics has revealed a rapidly growing number of metabolites that show concentration alterations in different cancers. The Human Metabolome Database (HMDB) keeps an up-to-date repository of cancer-associated metabolites and their roles in different cancer types [19].

The objective of this work is to identify perturbed metabolic processes characteristic of breast cancer cell lines of the subtypes ER(+), PR(+), and HER2(3+), which could form the basis of the identification of new markers for diagnosis and drug targets.

## 2. Results

### 2.1. Clustering of Cell Types Based on Metabolite Concentrations

First of all, we assessed whether the panel of 33 internal metabolites, quantified using the described analytical pipeline, can be used to cluster different cell types. The metabolite concentration data, in relative area units, are available in Supplementary File S1.

**Figure 1:**
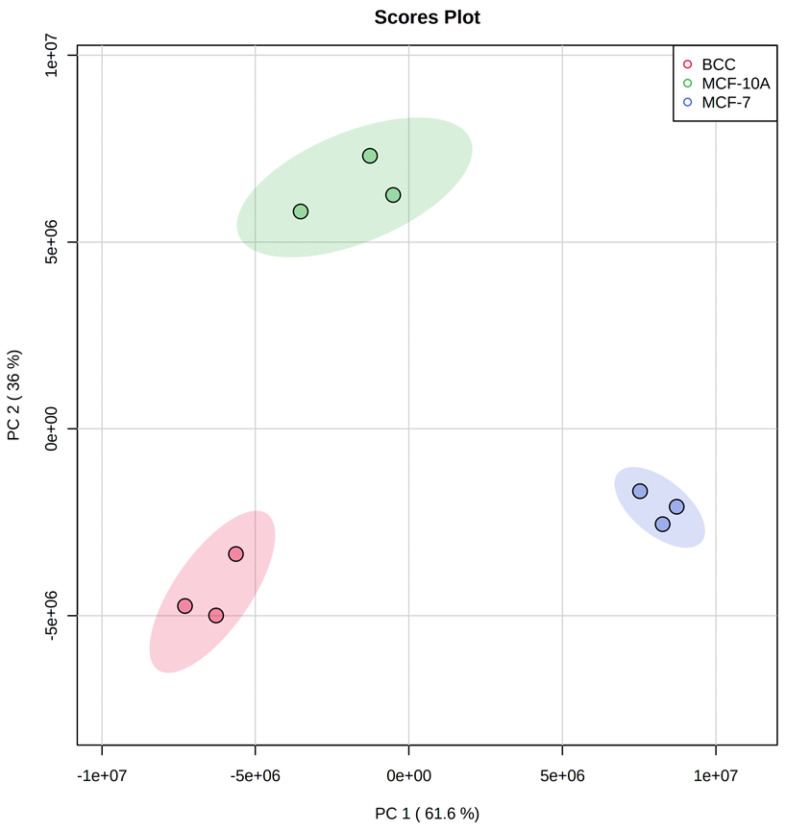
PCA plot showing the differences between the three tested cell lines. Shadowed areas show 95% confidence ellipses.

Principal Component Analysis (PCA) was carried out (Figure 1) and showed that 97.6% of the total variability in the data can be assigned to the first two principal components, with the first component accounting for 61.6% of the variability and the second for 36%. As shown in Figure 1, the second component clearly separates the non-malignant cell line MCF-10A from MCF-7 and BCC, while the first component captures variation across the three cell lines.

### 2.2. Clustering of Metabolites Based on Their Concentration Patterns

**Figure 2:**
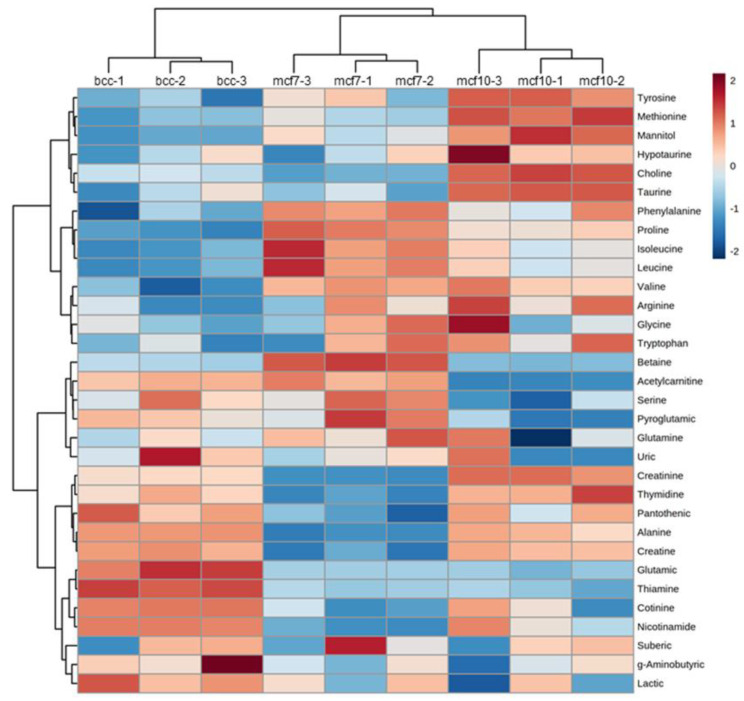
Heat map showing the relative abundances of each metabolite in each sample. The dendrograms show the results of hierarchical clustering, which aims to identify groups of metabolites with similar concentration patterns across samples.

The results of PCA indicate that 36% of the variation in metabolite concentrations observed between the three cell lines is associated with malignancy. In order to identify the metabolites that contribute the most to separating the non-malignant MCF-10A cell line from the two others, the data are represented in the form of a heat-map (Figure 2). The six metabolites in the upper cluster of the graph (tyrosine, methionine, mannitol, hypotaurine, choline and taurine) are characterized by a higher concentration in MCF-10A compared to MCF-7 and BCC. Four metabolites (betaine, acetyl-carnitine, serine and pyroglutamic acid) form a cluster characterized by higher concentrations in MCF-7 and BCC compared to MCF-10A.

As discussed later, six perturbed metabolites are related to the methionine cycle or have homocysteine (an intermediate of the methionine cycle) as a metabolic precursor. These metabolites are: methionine, taurine, hypotaurine, choline, betaine and pyroglutamic acid.

**Figure 3:**
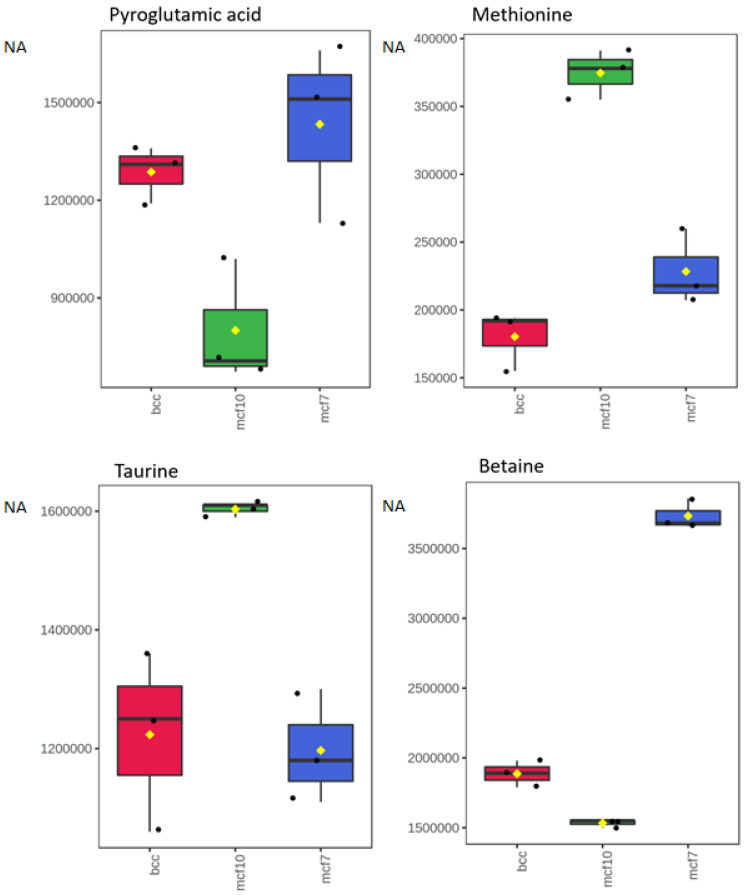
Box plots showing the concentration distributions of pyroglutamic acid, methionine, taurine and betaine in each of the three studied cell lines. The concentrations are not absolute, and reflect normalized areas (NA) of the corresponding peaks in the chromatogram.

Box plots showing the concentration distribution of some of the discussed metabolites are shown in Figure 3.

### 2.3. Differential Gene Expression of the Three Breast-Derived Cell Lines

**Figure 4:**
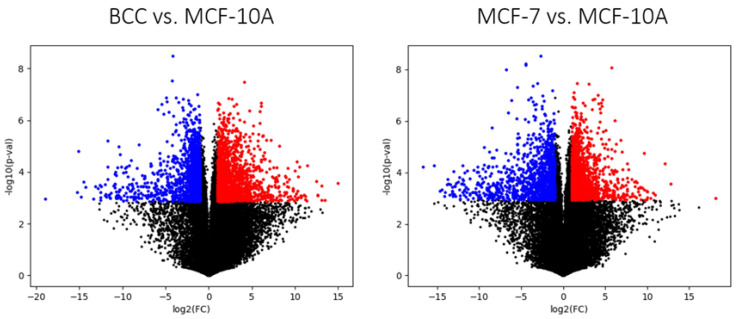
Volcano plots showing the distribution of up-regulated (red) and down-regulated (blue) genes and genes with no differential expression (black) in cancer cells vs. MCF-10A. The selected cut-off values to select differentially expressed genes were a false discovery rate of 0.01 and a fold-change of 2.

Differentially expressed genes were identified using a false discovery rate of 0.01 and a fold change of 2 as cut-off values. A total of 1495 genes showed significant up-regulation in MCF-7 compared MCF-10A and 1335, in which they were significantly down-regulated. For BCC, 1926 genes were up-regulated and 1669 down-regulated. Among the up-regulated genes, 274 were common to both cancer cell lines, and 563 of the down-regulated genes were observed in both cancer cell lines. The complete list of differentially expressed genes can be found in Supplementary File S2. Volcano plots summarizing the results of differential expression analysis are shown in Figure 4. A KEGG pathway enrichment test was carried out using webgestalt.org. No significantly enriched pathways among the consensus up-regulated genes (with a cut-off false discovery rate of 0.05). Among the consensus down-regulated genes, 10 KEGG pathways were identified (see Supplementary Figure S1). Half of these pathways are associated with the response to pathologies, and the rest are related to signaling receptors, such as IL-17 signaling, or cell adhesion pathways. In general, this reveals that cancer cells tend to become insensitive to extracellular signaling and detach from the extracellular matrix. No metabolic processes, which are the focus of this article, were identified with this kind of gene set analysis, which stresses the need to use GSMMs as scaffolds for the analysis and interpretation of transcriptomic data.

Among the 274 genes up-regulated in both cancer cell lines, 44 were metabolic genes, involved in 131 metabolic reactions. Among the 663 consensus down-regulated genes, 130 were metabolic genes, which are associated with 832 metabolic reactions. The large number of reactions affected by the down-regulated genes comes from the fact that many of these down-regulated genes are involved in cross-membrane transport. For example, the solute carrier family 36 member 4 gene, SLC36A4 (ENSG00000180773), is involved in four transport reactions (of glycine, alanine, proline and serine). The transporter SLC6A15, also down-regulated in both cancer cell lines, is involved in the transport of 11 amino acids. A full list of the metabolic reactions linked to up- and down-regulated genes can be found in Supplementary File S3. In this paper, we aim to find correlations between transcriptomics and metabolomics, so in further sections, we will focus on finding relationships between changes at the transcriptomic and metabolomic levels.

### 2.4. Relationships between Perturbed Metabolites and Expression of Metabolic Genes

Among the perturbed metabolites that were identified in the previous analysis, three of them (methionine, taurine and hypotaurine) can be traced back to a common metabolic precursor (homocysteine), which is an intermediate in the methionine cycle. Pyroglutamic acid is formed from the break-down of glutathione, which also can be traced back to homocysteine; however, its carbon atoms come from glutamine, which was incorporated during the synthesis of glutathione.

The gene AHCY, coding the enzyme adenosylhomocysteinase, which breaks down S-adenosylhomocysteine into adenosine and l-homocysteine, showed significantly lower expression in MCF-7 and BCC cell lines compared to MCF-10A. AHCY has been previously identified as a tumor suppressor gene involved in p53-induced cell cycle arrest [21]. Together with the lower concentration of methionine, the decreased expression of AHCY in malignant cell lines suggests lower activity of the methionine cycle.

**Figure 5:**
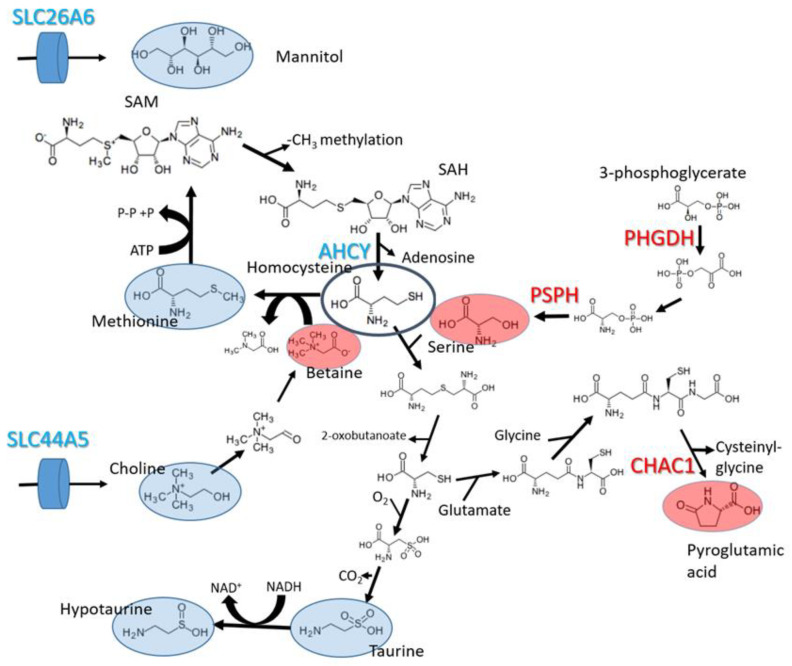
Perturbed metabolic processes identified through the integrated analysis of metabolomics and transcriptomics. Metabolites and genes with lower concentrations and expression levels in malignant cells are marked in blue, and metabolites with higher concentrations and genes with higher expression levels in malignant cells are marked in red.

Two more of the perturbed metabolites are linked to the methionine cycle (choline and betaine). As shown in Figure 5, betaine is synthesized from choline (a vitamin obtained from the diet) and provides methyl groups to the methionine cycle, which are used in DNA and protein methylation, using S-adenosyl methionine (SAM) as a methyl donor [22]. The internal pool of choline appeared to be smaller in both cancer cell lines compared to MCF-10A. This could be explained by the down-regulation of SLC44A5, which codes a choline transporter. Adversely, betaine shows a higher concentration in malignant cell lines. Betaine (produced from choline) can only be consumed by betaine-homocysteine methyl transferase, a step in the methionine cycle. The lower activity of the methionine cycle can explain the reduced consumption rate of betaine, resulting in its accumulation within the cells.

The RNA-seq data revealed that two genes involved in serine biosynthesis from the glycolytic intermediate 3-phosphoglycerate (PHGDH and PSPH) are more expressed in the MCF-7 and BCC cell lines compared to MCF-10A. The gene PHGDH codes phosphoglycerate dehydrogenase and has been shown to be overexpressed in several cancers [23]. It has been shown [24] that the overexpression of PHGDH in the cell line MCF-10A causes phenotypic alterations that predispose the cells to malignant transformation.

Pyroglutamic acid is a metabolic dead end that results from the degradation of glutathione. Our transcriptomic analysis revealed the overexpression of the gene CHAC1 in both cancer cell lines, which show increased intracellular pools of pyroglutamic acid. The function of this gene product in breaking down glutathione into cysteinyl-glycine and pyroglutamic acid was characterized in 2012 [25]. The knock-down of this gene has been shown to reduce cell migration and proliferation, while its overexpression has the opposite effect. The expression level of CHAC1 is also correlated with a more advanced stage and poorer prognosis in breast and ovarian tumors [26].

Other perturbed metabolites are metabolically unrelated to the previously discussed metabolic processes. For example, increased acetyl-carnitine points to higher activity of the carnitine shuttle, which is responsible for the transfer of fatty acids from the cytosol to the mitochondria. The relationship between the carnitine shuttle and cancer is well established [27]. Finally, the lower concentrations of mannitol can only be explained by the lower activity of transporters, as mannitol is a metabolically inert compound in humans. Indeed, the gene SLC26A6, which codes a mannitol transporter, has lower expression levels in both BCC and MCF-7 compared to MCF-10A.

**Figure 6:**
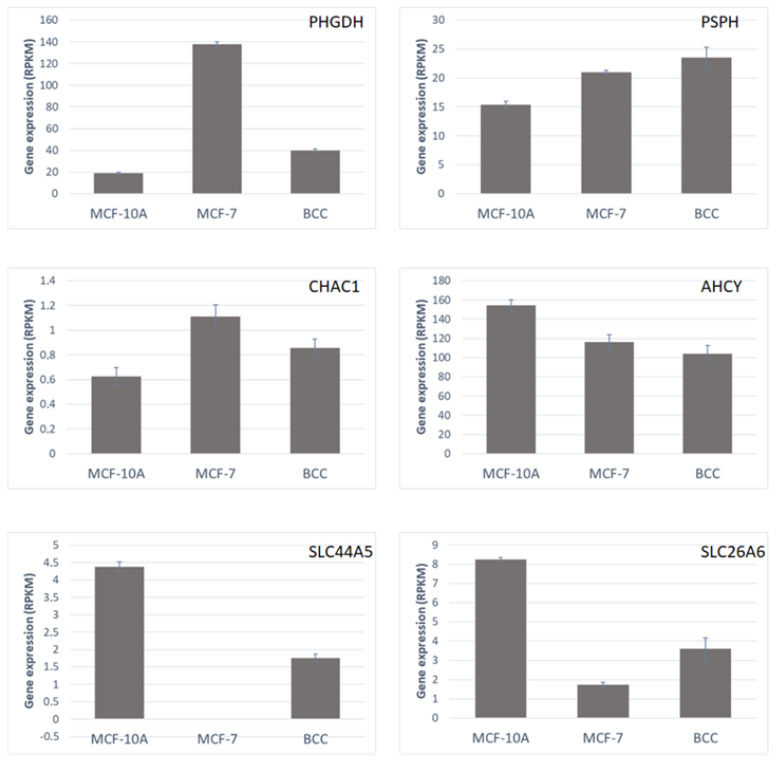
Expression levels, in each of the three studied cell lines, of metabolic genes that are directly linked to the observed perturbations in intracellular metabolic pools, in reads per kilobase per million reads (RPKM). Error bars represent standard deviations (*n* = 3).

The expression levels of genes that could be directly linked to perturbations in intracellular metabolic pools are shown in Figure 6.

### 2.5. Results of Flux Balance Analysis (FBA)

The obtained gene expression data were used to constrain a genome-scale metabolic model using the python library pyTARG [10]. FBA was used to obtain predictions of the distribution of metabolic fluxes in each of the studied cell lines. The most relevant observation was clear down-regulation of the respiratory chain in cancer cell lines, which is consistent with Warburgs’s effect and had been previously observed in a large panel of different cancer cell lines [10]. The reaction rate of ATP-synthase decreased in both cancer cells, and the genes ATP1B3 and ATP6V1H were down-regulated. Complex III of the respiratory chain also showed a lower rate, concomitantly with the down-regulation of two of the genes involved: UQCRH and UQCR1. Complex II of the respiratory chain also showed a lower metabolic flux, as a result of the down-regulation of the gene NDUFB8. Extracellular serine uptake was also lower in both malignant cell lines, as a result of the down-regulation of SLC36A4, which codes a serine/proton symporter. Lower transformation rates of CO_2_ into bicarbonate are also predicted in malignant cells, as a result of the down-regulation of two carbonic anhydrases (CA6 and CA2). A list of the perturbed metabolic reactions and their associated genes can be found in Supplementary File S4.

## 3. Discussion

There is high metabolic heterogeneity between different breast cancer cell lines, even between those belonging to the same molecular sub-types, (ER(+), PR(+), and HER2(3+)). However, some internal metabolites can be linked to malignancy. Transcriptomic data allowed us to establish relationships between the observed perturbations in internal metabolites and changes in the expression levels of key enzymes. 

Two main conclusions could be obtained from the integrated analysis of transcriptomic and metabolomic data. First of all, the MCF-7 and BCC cell lines showed lower internal methionine, taurine and hypotaurine levels, which, together with the down-regulation of AHCY (adenosylhomocysteinase), reveals decreased activity of the methionine cycle in cancer cells. On the other hand, a higher concentration of serine and higher expression of the genes PSPH and PHGDH are consistent with higher serine intracellular biosynthesis (which compensates for the lower extracellular serine uptake caused by the down-regulation of SLC36A4). Both methionine and serine are closely related to one-carbon metabolism. 

The methionine cycle is involved in the regeneration of the methyl donor SAM, while serine is involved in the regeneration of 5-methyl-tetrahydrofolate. SAM acts within the cell as a donor of methyl groups for the methylation of histones and DNA. High SAM concentrations have been shown to have anti-cancer effects (promoting apoptosis or down-regulating oncogenes through methylation); in particular, this effect has been shown on the MCF-7 cell line [28]. 5-methyl-tetrahydrofolate, which uses serine instead of betaine as a donor of methyl groups, is involved in purine biosynthesis. The relationship between one-carbon metabolism and cancer is not new [23]. Indeed, the relationship between dietary folate and cancer development was already noticed in the 1940s, and led to the discovery of the anticancer drugs known as antifolates [29]. More recently, other clinical implications of intracellular serine synthesis have been discovered. For example, serine synthesis has been shown to be activated by the G9A epigenetic program, and G9A inhibitors, such as BIX01294, can exhibit anti-cancer activity [30]. 

Besides its role as methyl donor, serine is also a precursor to the synthesis of glycine and other compounds, such as glutathione, involved in cellular resistance to oxidative stress. Serine can be synthesized de novo or uptaken from the exterior, and both processes have been shown to have an impact on cancer proliferation [24,31]. Serine and glycine starvation have been shown to decrease tumor growth and improve survival [32]. RNA-seq data indicate that MCF-7 and BCC cell lines both rely on de novo synthesis to keep a high intracellular concentration of serine. This feature of relying on intracellular synthesis instead of importing extracellular metabolites has also been observed in the case of cholesterol [17]. The reliance of cancer cells on the de novo synthesis of serine makes enzymes involved in serine biosynthesis potential drug targets. Protein kinase C zeta (PKCζ) inhibits the transcription of PHGDH and PSAT1 and also phosphorylates PHGDH, decreasing its catalytic activity [33]. Other small molecules inhibiting PHGDH have proven effective at reducing cell proliferation rates in vitro, and also xenografts in vivo [34,35].

## 4. Materials and Methods

### 4.1. Cell Lines and Culture Medium

MCF-7 (human breast adenocarcinoma cell line; CLS—Cell Lines Service, Eppelheim, Germany) and BCC cells [11] were cultured in Dulbecco’s Modified Eagle Medium: Ham’s F-12 (1:1; DMEM/F-12) (Life technologies, Carlsbad, CA, USA) medium, supplemented with 10% fetal bovine serum (FBS) (Sigma-Aldrich, St. Louis, MO, USA), 100 U/mL penicillin and 100 µg/mL streptomycin (pen-strep) (Sigma-Aldrich, St. Louis, MO, USA). MCF-10A cells (ATCC, Wesel, Germany) were cultured in DMEM/F-12, supplemented with 5% donor equine serum (GE Healthcare Life Sciences, Logan, UT, USA), pen-strep, 10 µg/mL human recombinant insulin (Life Technologies, Carlsbad, CA, USA), 100 ng/mL cholera toxin (Sigma-Aldrich, St. Louis, MO, USA), 20 ng/mL recombinant human epidermal growth factor (Thermo Fisher Scientific, Frederick, MD, USA) and 500 ng/mL hydrocortisone (Sigma-Aldrich, St. Louis, MO, USA). Cells were maintained at 37 °C in a humidified incubator with 5% CO_2_. Cells were incubated for 24 h. Metabolite extraction was performed as described earlier by Sellick and co-workers [36]. 

### 4.2. UPLC-ESI-MS Conditions

The samples were analyzed following the protocol published by Virgiliou and co-workers [37], which allowed su to identify 100 different polar compounds. The Acquity H-Class UPLC system (Waters, Milford, MA, USA) was used to analyze polar compounds in cell extracts. The chromatograph was equipped with a YMC-Triart C18 (100 × 2.0 mm, 1.9 µm) column (YMC, Kyoto, Japan). Mass spectrometry (MS) data were obtained in negative mode in the 50 *m*/*z* to 250 *m*/*z* range with a Xevo TQD triple quadrupole tandem mass spectrometer (Waters, Milford, MA, USA) coupled with an electrospray ionization (ESI) ion source. The column was set at 40 °C. Elution was applied with a mobile phase of 0.1% formic acid water solution (solvent A) and acetonitrile (solvent B). Flow rate was set to 0.4 mL/min. The starting conditions were set to 95% of solvent A. Gradient elution was applied with the following proportions of solvent A: 0 to 0.2 min was set to 95%, 0.2 to 1.5 min was set to 10%, 1.5 min to 1.8 min was maintained at 90%, and 1.8 to 2 min was set back to the starting conditions. The settings for the mass spectrometer were as follows: capillary voltage was set to negative 2 kV, source temperature to 150 °C, desolvation gas (nitrogen) temperature to 400 °C, gas flow to 700 L/h and cone gas flow to −20 L/h. Cone voltage was set to 25 V.

### 4.3. Analysis of Metabolomics Data

Based on the retention times reported by Virgiliou and co-workers [37,38] different metabolites were identified, which were present in all the samples. Three biological replicates were carried out. The data were further analyzed using MetaboAnalyst [33] for the statistical tests, and the plots are discussed in the following section. Pathway enrichment with MetaboAnalyst did not allow us to identify clear metabolic relationships between perturbed metabolites (beyond the obvious ones such as taurine and hypotaurine). 

### 4.4. RNA Sequencing

Three biological replicates from each of the three cell lines (MCF-7, MCF-10A and BCC) were sent to Zymo Research for next-gen RNA sequencing. The samples were prepared for shipment using DNA/RNA Shield^TM^ (Zymo Research, Irvine, CA USA) by mixing 1 million cells with 500 µL of Shield stabilization solution. The company constructed RNA-Seq libraries from 500 ng of total RNA using the Zymo-Seq RiboFree Total RNA Library Prep Kit (Zymo Research, Irvine, CA, USA) according to the manufacturer’s instructions, before sequencing on an Illumina NovaSeq (Illumina, San Diego, CA, USA) to a sequencing depth of at least 30 million read pairs (150 bp paired-end sequencing) per sample.

### 4.5. Analysis of RNA-Seq Data

The obtained pair-ended reads were aligned on a reference sequence (the complete list of human transcripts was obtained from Ensembl BioMart) using Bowtie2 [39]. The resulting SAM files were analyzed using customized python scripts based on the HTSeq library [40] (provided in the Supplementary File Code1). The expression of each gene was calculated in reads per kilobase per million reads (RPKM). Student’s *t*-test was used to identify up-regulated and down-regulated genes comparing malignant and non-malignant cell lines. The obtained values were corrected for multiple testing as described in previous work [11] (the python code used for the differential expression analysis is provided in the Supplementary File Code2). Raw and processed data were submitted to NCBI’s Gene Expression Omnibus (GEO) database with the accession number GSE223718.

### 4.6. Integrated Data Analysis Using pyTARG

In order to obtain meaningful relationships between metabolomics and transcriptomics, we used a GSMM [10] in order to correlate the observed perturbations in metabolite levels with concomitant changes in gene expression. The python library pyTARG, developed previously [10], was used to estimate metabolic fluxes from the obtained gene expression data. The pyTARG library works as follows: Each of the reactions in the model, which are catalyzed by metabolic enzymes, is bound to have a maximal rate proportional to the expression level of the gene coding the enzyme (which is obtained from RNA-seq data). Once the model is constrained, a metabolic flux distribution is simulated by optimizing the rate of biomass production. As there are three experimental replicas for each cell line, three metabolic flux distributions are obtained, which enables the identification of reactions that have statistically different rates in different cell lines. Metabolic reactions that were differentially used between malignant and non-malignant cells were identified using a *t*-test and a false discovery rate of 0.05 after correction for multiple testing. It was also required to have an average difference in flux higher than 0.001 mmol h^−1^ g-DW^−1^ (millimoles per hour per gram of dry weight). It is known that broad changes in metabolic flux distributions are typically driven by changes in the expression levels of a relatively small number of metabolic genes. In order to identify these flux-controlling genes, reactions showing a significant change in both the estimated reaction rate and the expression level of at least one of their associated genes were identified.

## 5. Conclusions

The described analytical pipeline allowed us to quantify enough metabolites to obtain a clear metabolic footprint of each of the tested cell lines. Among the identified metabolites, 10 of them appeared to be associated with malignancy. The intracellular concentration of methionine was significantly lower in both malignant cell lines compared to the non-malignant MCF-10A. Together with the down-regulation of the metabolic gene AHCY and the depletion of taurine and hypotaurine (which originate from homocysteine), this observation suggests lower activity of the methionine cycle in cancer cell lines, which would cause a lower re-methylation rate of SAM. The pro-apoptotic effects of SAM supplementation in breast cancer cells have already been shown. Our data suggest that the supplementation of methionine and betaine, leading to higher flux in the methionine cycle and the higher production of SAM, could also have the same effects. This is an interesting hypothesis that should be tested in the future. An increased intracellular pool of serine was observed, which is consistent with the increased intracellular synthesis rate caused by the over-expression of the genes PHGDH and PSPH. The targeting of serine biosynthesis could be a potential alternative for the treatment of breast cancer. Finally, the recently discovered relationship between the gene CHAC1 and the production of pyroglutamic acid was confirmed. The results of this work point to a possible role of pyroglutamic acid as a biomarker, and an oncogenic role of the gene CHAC1, which remains to be explored further. All the presented conclusions are based on two breast cancer cell lines, and whether they apply to other cell lines and cancer types remains to be tested.

## Data Availability

Raw and processed data were submitted to NCBI’s Gene Expression Omnibus (GEO) database with the accession number GSE223718.

## Supplementary Materials

The following supporting information can be downloaded at: https://www.mdpi.com/article/10.3390/molecules28114535/s1, Supplementary File S1: Metabolite concentration data, in relative area units; Supplementary File S2: Differentially expressed genes; Supplementary File S3: Differentially expressed metabolic genes and their associated reactions; Supplementary File S4: Perturbed metabolic fluxes (predicted by pyTARG) and their associated genes.

## Sample Availability

The datasets used and/or analyzed during the current study are available from the corresponding author on reasonable request.

## References

[1] Warburg O., Wind F., Negelein E. (1927). The Metabolism of tumors in the body. J. Gen. Physiol..

[2] Hanahan D., Weinberg R.A. (2011). Hallmarks of cancer: The next generation. Cell.

[3] Hanahan D. (2022). Hallmarks of Cancer: New Dimensions. Cancer Discov..

[4] Dang C.V. (2012). Links between metabolism and cancer. Genes Dev..

[5] Seyfried T.N., Shelton L.M. (2010). Cancer as a metabolic disease. Nutr. Metab..

[6] Vander Heiden M.G. (2011). Targeting cancer metabolism: A therapeutic window opens. Nat. Rev. Drug Discov..

[7] Ward P.S., Thompson C.B. (2012). Metabolic reprogramming: A cancer hallmark even Warburg did not anticipate. Cancer Cell.

[8] Thiele I., Swainston N., Fleming R.M., Hoppe A., Sahoo S., Aurich M.K., Haraldsdottir H., Mo M.L., Rolfsson O., Stobbe M.D. (2013). A community-driven global reconstruction of human metabolism. Nat. Biotechnol..

[9] Raškevičius V., Mikalayeva V., Antanavičiūtė I., Ceslevičienė I., Kairys V., Bordel S. (2018). Genome scale metabolic models as tools for drug design and personalized medicine. PLoS ONE.

[10] Bordel S. (2018). Constraint based modeling of metabolism allows finding metabolic cancer hallmarks and identifying personalized therapeutic windows. Oncotarget.

[11] Antanavičiūtė I., Mikalayeva V., Ceslevičienė I., Milašiūtė G., Skeberdis V.A., Bordel S. (2017). Transcriptional hallmarks of cancer cell lines reveal an emerging role of branched chain amino acid catabolism. Sci. Rep..

[12] Bordel S., Agren R., Nielsen J. (2010). Sampling the solution space in genome-scale metabolic networks reveals transcriptional regulation in key enzymes. PLoS Comput. Biol..

[13] Borgos S.E., Bordel S., Sletta H., Ertesvag H., Jakobsen O., Bruheim P., Ellingsen T.E., Nielsen J., Valla S. (2013). Mapping global effects of the anti-sigma factor MucA in Pseudomonas fluorescens SBW25 through genome-scale metabolic modeling. BMC Syst. Biol..

[14] Martínez J.L., Bordel S., Hong K., Nielsen J. (2014). Gcn4p and the Crabtree effect of yeast: Drawing the causal model of the Crabtree effect in Saccharomyces cerevisiae and explaining evolutionary trade-offs of adaptation to galactose through systems biology. FEMS Yeast Res..

[15] Bordel S., Nielsen J. (2010). Identification of flux control in metabolic networks using non-equilibrium thermodynamics. Metab. Eng..

[16] Mikalayeva V., Ceslevičienė I., Sarapinienė I., Žvikas V., Skeberdis V.A., Jakštas V., Bordel S. (2019). Fatty acid synthesis and degradation interplay to regulate the oxidative stress in cancer cells. Int. J. Mol. Sci..

[17] Mikalayeva V., Pankevičiūtė M., Žvikas V., Skeberdis V.A., Bordel S. (2021). Contribution of branched chain amino acids to energy production and mevalonate synthesis in cancer cells. Biochem. Biophys. Res. Commun..

[18] Antoniewicz M.R., Kelleher J.K., Stephanopoulos G. (2007). Elementary metabolic units (EMU): A novel framework for modelling isotopic distributions. Metab. Eng..

[19] Wishart D.S., Mandal R., Stanislaus A., Ramirez-Gaona M. (2016). Cancer Metabolomics and the Human Metabolome Database. Metabolites.

[20] Ward P.S., Patel J., Wise D.R., Abdel-Wahab O., Bennett B.D., Coller H.A., Cross J.R., Fantin V.R., Hedvat C.V., Perl A.E. (2010). The common feature of leukemia-associated IDH1 and IDH2 mutations is a neomorphic enzyme activity converting alpha-ketoglutarate to 2-hydroxyglutarate. Cancer Cell.

[21] Leal J.F., Ferrer Y., Blanco-Aparicio C., Hernandez-Losa J., Ramon y Cajal S., Carnero A., Lleonart M.E. (2008). S-adenosylhomocysteine hydrolase downregulation contributes to tumorigenesis. Carcinogenesis.

[22] Zhao G., He F., Wu C., Li P., Li N., Deng J., Zhu G., Ren W., Peng Y. (2018). Betaine in Inflammation: Mechanistic Aspects and Applications. Front. Immunol..

[23] Amelio I., Cutruzzolá F., Antonov A., Agostini M., Melino G. (2014). Serine and glycine metabolism in cancer. Trends Biochem. Sci..

[24] Locasale J.W., Grassian A.R., Melman T., Lyssiotis C.A., Mattaini K.R., Bass A.J., Heffron G., Metallo C.M., Muranen T., Sharfi H. (2011). Phosphoglycerate dehydrogenase diverts glycolytic flux and contributes to oncogenesis. Nat. Genet..

[25] Kumar A., Tikoo S., Maity S., Sengupta S., Sengupta S., Kaur A., Kumar Bachhawat A. (2012). Mammalian proapoptotic factor ChaC1 and its homologues function as gamma-glutamyl cyclotransferases acting specifically on glutathione. EMBO Rep..

[26] Goebel G., Berger R., Strasak A.M., Egle D., Muller-Holzner E., Schmidt S., Rainer J., Presul E., Parson W., Lang S. (2012). Elevated mRNA expression of CHAC1 splicing variants is associated with poor outcome for breast and ovarian cancer patients. Br. J. Cancer.

[27] Console L., Scalise M., Mazza T., Pochini L., Galluccio M., Giangregorio N., Tonazzi A., Indiveri C. (2020). Carnitine Traffic in Cells. Link With Cancer. Front. Cell Dev. Biol..

[28] Delle Cave D., Ilisso C.P., Mosca M., Pagano M., Martino E., Porcelli M., Cacciapuoti G. (2017). The Anticancer Effects of S-Adenosylmethionine on Breast Cancer Cells. JSM Chem..

[29] Newman A.C., Maddocks O.D.K. (2017). One-carbon metabolism in cancer. Br. J. Cancer.

[30] Chen W.L., Sun H.P., Li D.D., Wang Z.H., You Q.D., Guo X.K. (2017). G9a-An appealing antineoplastic target. Curr. Cancer Drug Targets..

[31] Possemato R., Marks K.M., Shaul Y.D., Pacold M.E., Kim D., Birsoy K., Sethumadhavan S., Woo H.K., Jang H.G., Jha A.K. (2011). Functional genomics reveal that the serine synthesis pathway is essential in breast cancer. Nature.

[32] Maddocks O.D., Berkers C.R., Mason S.M., Zheng L., Blyth K., Gottlieb E., Vousden K.H. (2013). Serine starvation induces stress and p53-dependent metabolic remodelling in cancer cells. Nature.

[33] Ma L., Tao Y., Duran A., Llado V., Galvez A., Barger J.F., Castilla E.A., Chen J., Yajima T., Porollo A. (2013). Control of nutrient stress-induced metabolic reprogramming by PKCζ in tumorigenesis. Cell.

[34] Ravez S., Spillier Q., Marteau R., Feron O., Frederick R. (2017). Challenges and opportunities in the development of serine synthetic pathway inhibitors for cancer therapy. J. Med. Chem..

[35] Pacold M.E., Brimacombe K.R., Chan S.H., Rohde J.M., Lewis C.A., Swier L.J., Possemato R., Chen W.W., Sullivan L.B., Fiske B.P. (2016). A PHGDH inhibitor reveals coordination of serine synthesis and one-carbon unit fate. Nat. Chem. Biol..

[36] Sellick C.A., Hansen R., Stephens G.M., Goodacre R., Dickson A.J. (2011). Metabolite extraction from suspension-cultured mammalian cells for global metabolite profiling. Nat. Protoc..

[37] Virgiliou C., Gika H.G., Theodoridis G.A. (2018). Metabolic Profiling: Methods and Protocols. Methods Mol. Biol..

[38] Pang Z., Chong J., Zhou G., Morais D., Chang L., Barrette M., Gauthier C., Jacques P.É., Li S., Xia J. (2021). MetaboAnalyst 5.0: Narrowing the gap between raw spectra and functional insights. Nucl. Acids Res..

[39] Langmead B., Salzberg S.L. (2012). Fast gapped-read alignment with Bowtie 2. Nat. Methods.

[40] Anders S., Pyl P.T., Huber W. (2015). HTSeq—A Python framework to work with high-throughput sequencing data. Bioinformatics.

