# Supplementary figures and images for: Multi-Omics Analysis Revealed Increased De Novo Synthesis of Serine and Lower Activity of the Methionine Cycle in Breast Cancer Cell Lines
Source: Molecules. 2023 Jun 3;28(11):4535. doi: 10.3390/molecules28114535 (PMC10254915; doi:10.3390/molecules28114535)

■ FDR ≤ 0.05    ■ FDR > 0.05

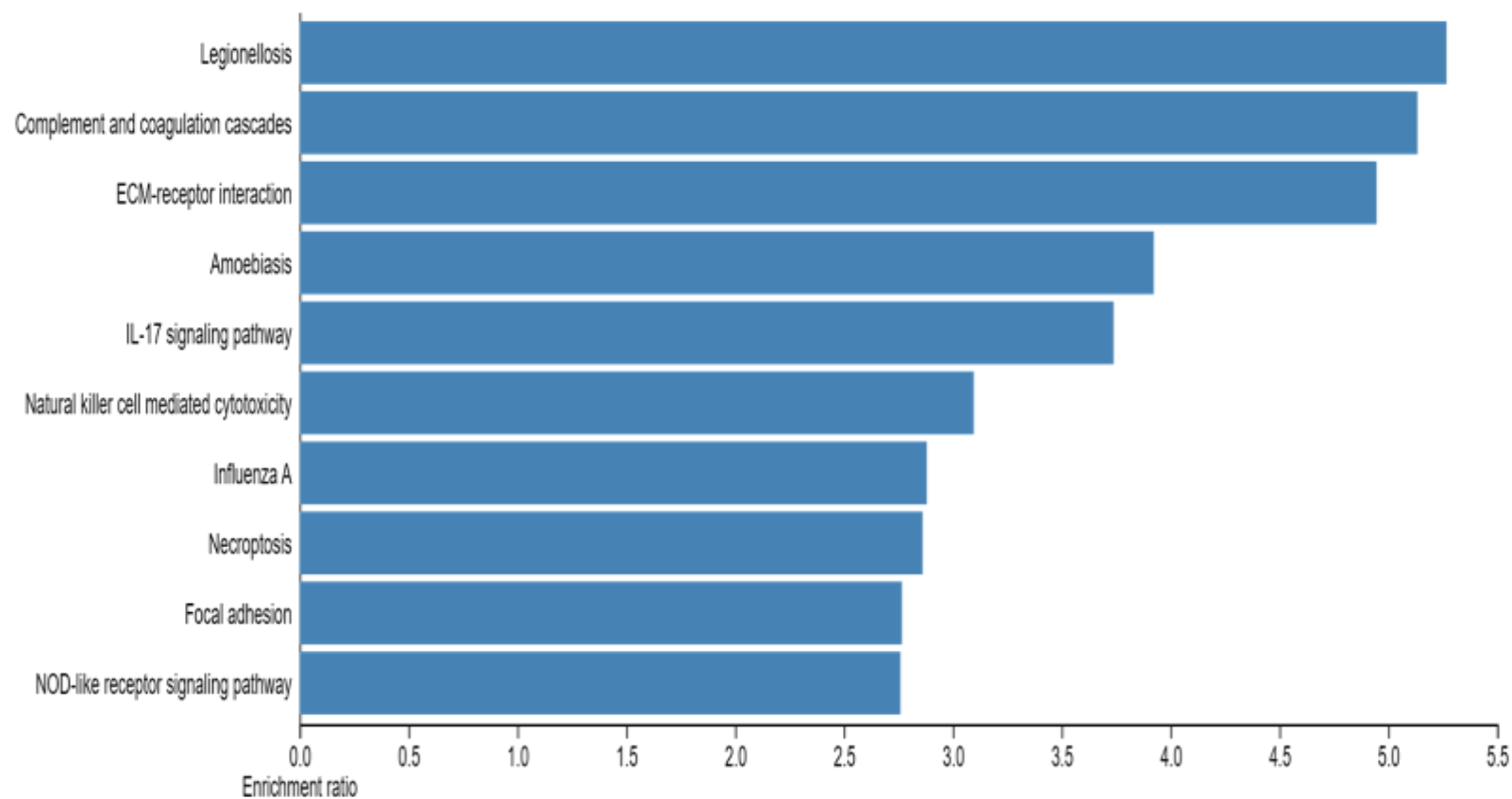

Supplement: Supplementary file 1 [file molecules-28-04535-s001.zip › Supplementary Figure S1.pdf]
